# Evaluation of hemorrhagic shock and fluid resuscitation in pigs using handless Doppler carotid artery ultrasound

**DOI:** 10.1007/s00068-024-02481-3

**Published:** 2024-03-01

**Authors:** Xiaoli Zhao, Wei Yuan, Shuo Wang, Junyuan Wu, Chunsheng Li

**Affiliations:** 1 Department of Emergency Medicine, Beijing Friendship Hospital, Capital Medical University, Beijing, 100050 China; 2 Department of Emergency Medicine, Beijing Chaoyang Hospital, Capital Medical University, Beijing, 100020 China; 3 Department of Infectious Diseases (Fever Clinic), Beijing Hospital of Traditional Chinese Medicine, Capital Medical University, Beijing, 100010 China

**Keywords:** Hemorrhagic shock, Fluid resuscitation, Hemodynamics, Ultrasound

## Abstract

**Objective:**

This study aimed to utilize a hemorrhagic shock pig model to compare two hemodynamic monitoring methods, pulse index continuous cardiac output (PiCCO) and spectral carotid artery Doppler ultrasound (CDU). Additionally, we sought to explore the feasibility of employing CDU as a non-invasive hemodynamic monitoring tool in the context of hemorrhagic shock and fluid resuscitation.

**Design:**

Animal experiments.

**Setting and subjects:**

Female pigs were selected, and hemorrhagic shock was induced by rapid bleeding through an arterial sheath.

**Interventions:**

Hemodynamic monitoring was conducted using both PiCCO and CDU during episodes of hemorrhagic shock and fluid resuscitation.

**Measurements and main results:**

Among the 10 female pigs studied, CDU measurements revealed a significant decrease in carotid velocity time integral (cVTI) compared to baseline values under shock conditions. During the resuscitation phase, after the mean arterial pressure (MAP) returned to its baseline level, there was no significant difference between cVTI and baseline values. A similar trend was observed for carotid peak velocity (cPV). The corrected flow time (FTc) exhibited a significant difference only at the time of shock compared to baseline values. In comparison to PiCCO, there was a significant correlation between cVTI and MAP (*r* = 0.616, *P* < 0.001), stroke volume (SV) (*r* = 0.821, *P* < 0.001), and cardiac index (CI) (*r* = 0.698, *P* < 0.001). The carotid Doppler shock index (cDSI) displayed negative correlations with MAP (*r* =  − 0.593, *P* < 0.001), SV (*r* =  − 0.761, *P* < 0.001), and CI (*r* =  − 0.548, *P* < 0.001), while showing a positive correlation with the shock index (SI) (*r* = 0.647, *P* < 0.001).

**Conclusions:**

Compared to PiCCO, CDU monitoring can reliably reflect the volume status of hemorrhagic shock and fluid resuscitation. CDU offers the advantages of being non-invasive, providing real-time data, and being operationally straightforward. These characteristics make it a valuable tool for assessing and managing hemorrhagic shock, especially in resource-limited settings.

## Introduction

Hemorrhagic shock (HS) is a life-threatening condition responsible for approximately 1.9 million deaths worldwide annually [1]. Moreover, survivors frequently experience poor functional outcomes and significantly heightened long-term mortality [2, 3]. This condition results in acute, substantial reductions in effective circulatory volume due to massive blood loss, leading to decreased venous return, cardiac output, and eventual decline in mean arterial pressure (MAP). Precise hemodynamic monitoring plays a pivotal role in accurately assessing changes in patient volume status and facilitating the treatment of hemorrhagic shock. The pulse index continuous cardiac output (PiCCO) represents a hemodynamic monitoring system that integrates various indices through intra-arterial and central venous catheterization. While PiCCO is recognized as a classic and precise standard for hemodynamic monitoring and has been extensively used in clinical practice, it is invasive and can only be employed within hospital settings, with the potential for adverse events such as infection and tissue damage [4]. It is now understood that a substantial number of trauma patients require reliable and dynamic hemodynamic assessments in non-hospital environments to determine the need for blood transfusion and fluid resuscitation. Hence, the ideal hemodynamic monitoring technology should be continuous, non-invasive, safe, feasible, and reliable. In recent years, non-invasive wearable hemodynamic monitoring devices, such as spectral carotid artery Doppler ultrasound (CDU), have been applied in various clinical scenarios, including cardiac arrest [5], hemodialysis [6], and gastrointestinal bleeding [7] patients. The waveform and parameter changes of CDU can predict the fluid response status with undifferentiated shock [8] and distinguish between pulseless electrical activity and restoration of spontaneous circulation during cardiopulmonary resuscitation [5]. Based on these studies, CDU has the potential to become a new method for monitoring whole volume status. However, no comparative study has hitherto been conducted between non-invasive wearable hemodynamic monitoring devices and invasive hemodynamic monitoring with PiCCO, and the reliability and sensitivity of this monitoring method remain unclear for acute volume changes like those observed during HS and fluid resuscitation.

To explore the feasibility of CDU monitoring and guiding the treatment of hemorrhagic shock, we established a pig model of HS to investigate changes in CDU parameters at various time points, from the onset of blood loss to the development of shock and subsequently during fluid resuscitation. And we compare these CDU parameters with traditional invasive PiCCO parameters to examine the correlation between them, thus providing a theoretical foundation for the clinical application of CDU.

## Materials and methods

### Animal preparation

We utilized ten healthy domestic female Beijing Landrace pigs (67 ± 1 days old, 34 ± 1 kg in weight), which were sourced from a registered laboratory animal center in Beijing, China. This study was carried out in strict adherence to the animal care and application guidelines established by the Animal Care and Use Committee of Capital Medical University (No.2022–04-054). Animal experiments followed the Guidelines for the Care and Use of Animals as articulated in the Declaration of Helsinki [9]. The animals were fasted overnight but had free access to water. They were initially sedated with intramuscular midazolam (2 mg/kg) and maintained under anesthesia with continuous intravenous propofol (2–3 mg/kg/hr). A cuffed 6.5 mm endotracheal tube was inserted into the trachea, and the animals were mechanically ventilated using a volume-controlled ventilator (Mindray SV300, Shenzhen, China) with a tidal volume of 10 ml/kg and FiO_2_ at 0.21. A 4-F arterial catheter was placed in the left femoral artery to measure MAP, while a 6-F arterial sheath was inserted in the right femoral artery to prevent rapid bleeding (about 33 ml/min). Additionally, a 5-F central venous catheter was inserted through the left femoral vein for volume resuscitation. Room temperature was maintained at 26 °C, and body temperature was sustained above 37 °C using a heating pad.

### Experimental protocol

Following the surgical procedures, the animals were allowed to stabilize for 30 min, and baseline data were recorded. Hemorrhagic shock was induced by rapid bleeding via the arterial sheath, with HS considered to have occurred when the cardiac index (CI) and MAP dropped below 40% of their respective baseline values [10]. The blood collected during hemorrhagic shock was stored in sterile bags (S-400, Sichuan Nightingale Biological Co. Ltd.). Fluid resuscitation commenced after 30 min of shock, with the animals being resuscitated using the shed blood and receiving a basal crystalloid infusion of 30 ml/kg/h. Fluid resuscitation continued until MAP and CI returned to their baseline values and remained stable for 2 h. No vasoconstrictor drugs were administered throughout the experimental process.

### Measurements

#### PiCCO hemodynamic parameters

**Fig. 1:**
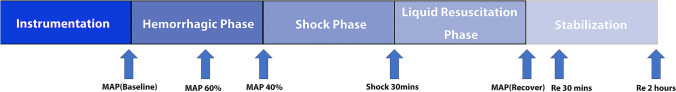
Experimental protocol and time points for collecting monitoring parameters

Arterial and central venous catheters were connected to an integrated bedside monitor (PiCCO; Pulsion Medical Systems, Munich, Germany) for continuous hemodynamic monitoring. Hemodynamic parameters, including heart rate (HR), MAP, CI, and stroke volume (SV), were measured at baseline, 60% of MAP, 40% of MAP (shock), 30 min after the onset of shock, MAP recovery, 30 min after recovery, and 2 h after recovery (Fig. 1).

#### CDU hemodynamic parameters

**Fig. 2:**
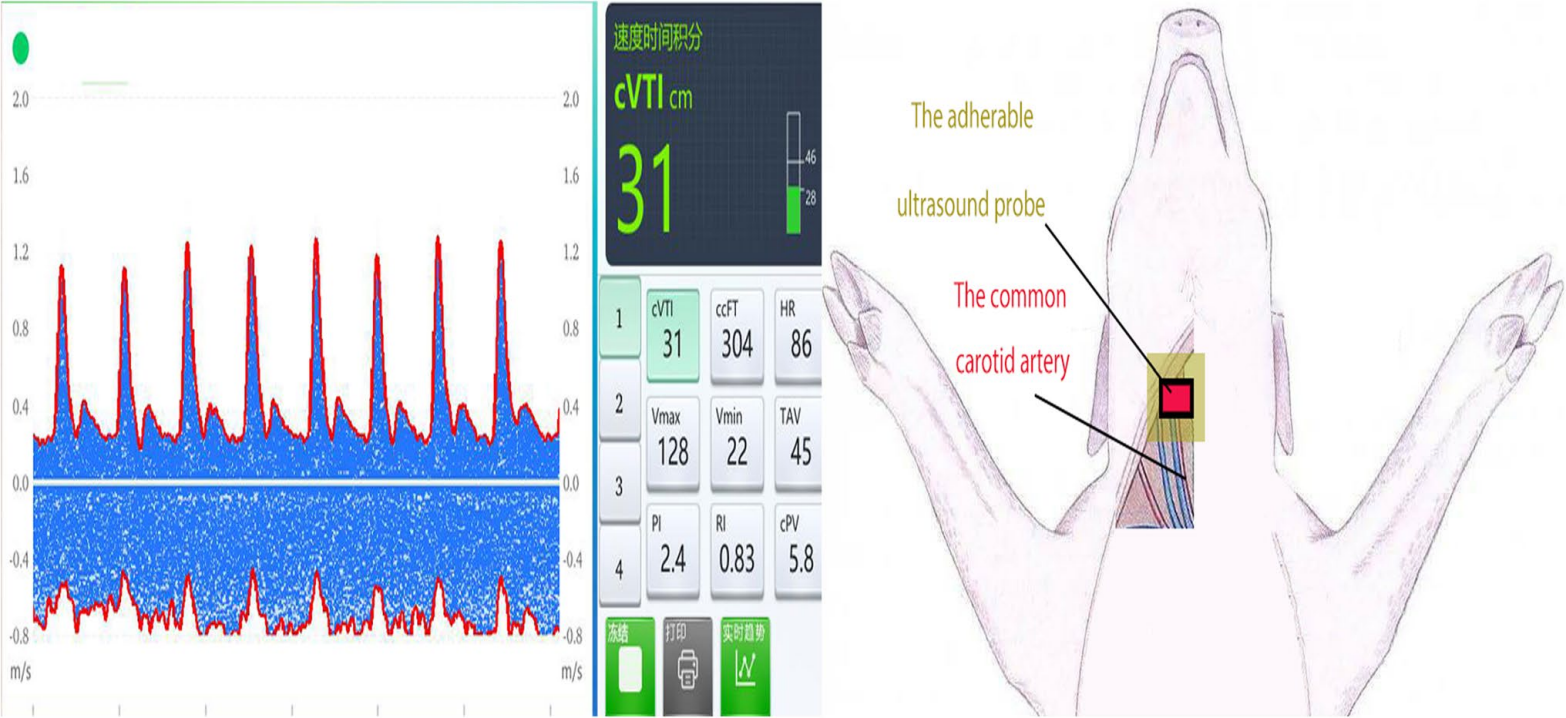
The screen display of CDU and ultrasound probe placement on the surface

**Fig. 3:**
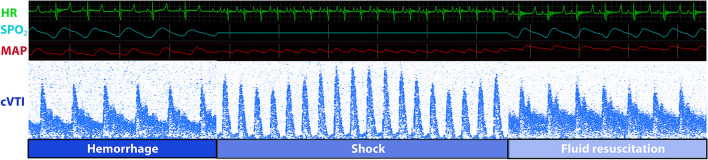
ECG monitoring and the cVTI curve from hemorrhage to fluid resuscitation. Abbreviations: HR, heart rate; SPO_2_, pulse oxygen saturation; MAP, mean arterial pressure; cVTI, carotid velocity time integral

Physiological parameters of carotid blood flow were recorded using an adherable ultrasound probe (Sensus Medical, Suzhou, China). After palpating the carotid pulse below the carotid bifurcation, the probe was positioned along the medial aspect of the sternocleidomastoid muscle above the common carotid artery (Fig. 2). The probe’s position was adjusted for optimal monitoring and then securely fixed in place. The probe was connected to a CAD FlowTM UCM Doppler Ultrasound System (Sensus Medical, Suzhou, China), which automatically monitored the velocity of the carotid pulse and calculated the carotid velocity time integral (cVTI), displayed on the system screen. The ultrasound system could also generate other parameters, including carotid pulse volume (cPV), flow time corrected (FTc), and carotid Doppler shock index (cDSI = HR/cVTI). In the CAD FlowTM UCM Doppler Ultrasound System, the maximum velocity at each time point in the waveform was estimated automatically, with the area under the curve representing the cVTI (Fig. 3). cPV was derived from cVTI and carotid cross-sectional area, while FTc was calculated using the formula: FTc = systolic flow time + (1.29 × (HR – 60)) [8].

### Statistical analysis

All statistical analyses were carried out using SPSS 24.0 software (SPSS, Chicago, IL, USA). Continuous variables were reported as mean ± standard deviation. The normal distribution and equality of variance for continuous variables were assessed using the Kolmogorov–Smirnov test and the homogeneity of variance test, respectively. Correlations were evaluated using Pearson correlation analysis, with a *P*-value less than 0.05 considered statistically significant.

## Results

### Baseline characteristics and amount of bleeding per pig

The ten female pigs utilized in this study had an average weight of 34 ± 1 kg and a mean age of 67 ± 1 days. The total blood loss per pig was 1019.80 ± 303.25 ml over a mean duration of 31.20 ± 12.76 min. The amount of bleeding and resuscitation time for each animal is detailed in Table 1. Blood loss volume is approximately 1.8 to 4.7% of animal weight. Hemodynamic parameters obtained from PiCCO measurements showed an HR of 115 ± 27 bpm, MAP of 108 ± 20 mmHg, CI of 4.24 ± 1.02 L/min, and SV of 36.00 ± 6.51 ml. Additionally, CDU measurements indicated that cVTI was 27.50 ± 9.33 cm, cPV was 14.50 ± 5.10 ml, FTc was 277.57 ± 38.01 ms under basal physiological conditions, and cDSI was 5.39 ± 2.41.

**Table 1:** The volume of blood and resuscitate time of each animal

Animal No	Weight(kg)	Age(d)	Blood volume removed (ml)	Resuscitate time
1	33	68	762 ml(within 28 min)	63 min
2	35	68	760 ml(within 20 min)	27 min
3	33	68	1538 ml(within 57 min)	39 min
4	35	68	1146 ml(within 36 min)	75 min
5	35	69	1419 ml(within 20 min)	52 min
6	33	67	1049 ml(within 30 min)	46 min
7	35	67	712 ml(within 49 min)	63 min
8	35	65	1073 ml(within 25 min)	43 min
9	35	67	1100 ml(within 28 min)	58 min
10	35	67	639 ml(within 19 min)	72 min

### Comparison of CDU parameters at different time points during hemorrhage and resuscitation

**Fig. 4:**
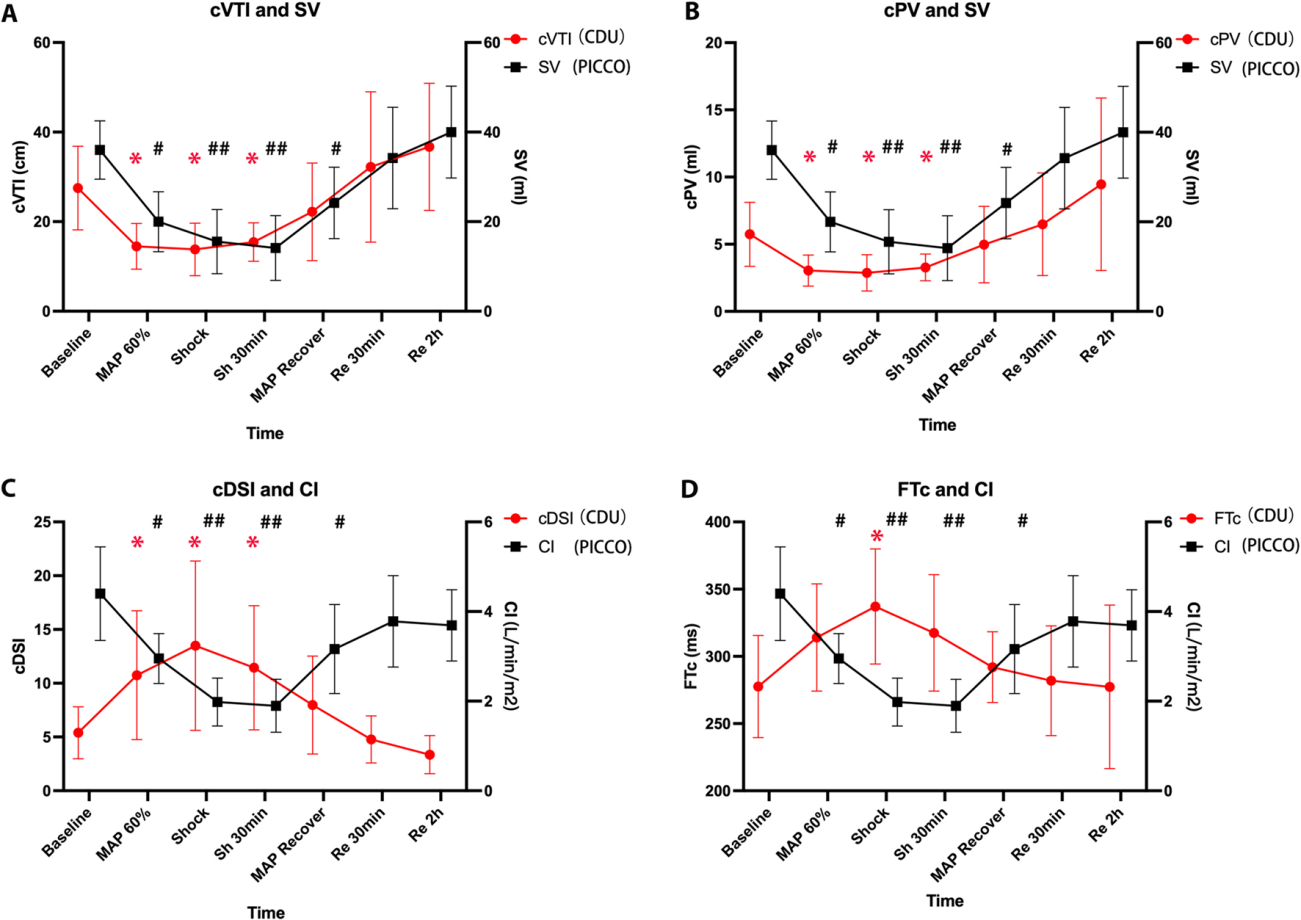
CDU and PiCCO parameters at different time points from hemorrhage to fluid resuscitation. **A** The trends of carotid velocity time integral (cVTI) and stroke volume (SV) changes. **B** The trends of carotid pulse volume (cPV) and SV changes. **C** The trends of carotid Doppler shock index (cDSI) and cardiac index (CI) changes. **D** The trends of corrected flow time (FTc) and CI changes. Abbreviations: MAP, mean arterial pressure. *Significantly different from baseline values, *P* < 0.05 (CDU parameters). #Significantly different from baseline values, *P* < 0.05 (PiCCO parameters). ##Significantly different from baseline values, *P* < 0.001

As shown in Fig. 4A–D, the MAP dropped to 60% of the baseline value, cVTI decreased to approximately 50% of the baseline value (cVTI _baseline_: 27.50 ± 9.34 cm vs. cVTI _60%MAP_: 14.50 ± 5.10 cm, *P* = 0.004). Under shock conditions, cVTI remained significantly lower than the baseline value. During the resuscitation phase, after MAP returned to its baseline level, there was no significant difference between cVTI and baseline values (cVTI _baseline_: 27.50 ± 9.34 cm vs. cVTI _recovery_: 22.20 ± 10.91 cm, *P* = 0.292).

cPV exhibited a consistent trend with cVTI. When MAP dropped to 60%, cPV significantly decreased compared to the baseline (cPV _baseline_: 5.74 ± 2.38 ml vs. cPV _60%MAP_: 3.04 ± 1.15 ml, *P* = 0.011). Until the resuscitation phase, with an increase in MAP, cPV gradually recovered (cPV _baseline_: 5.74 ± 2.38 ml vs. cPV _recovery_: 4.97 ± 2.85 ml, *P* = 0.537).

Correspondingly, since MAP decreased to 60%, cDSI remained higher than the baseline state under shock conditions (cDSI _baseline_: 5.39 ± 2.41 vs. cDSI _60%MAP_: 10.75 ± 5.99, *P* = 0.034). After recovery, cDSI returned to its basic level relatively (cDSI _baseline_: 5.39 ± 2.41 vs. cDSI _recovery_: 7.97 ± 4.56, *P* = 0.169).

FTc showed significant differences compared to the baseline value only at the time of shock (FTc _baseline_: 277.57 ± 38.01 ms vs. FTc _shock_: 317.48 ± 43.29 ms, *P* = 0.005).

Analysis of PiCCO parameters revealed that with a decrease in MAP, SV and CI remained below the baseline value (SV baseline: 36.00 ± 6.51 ml vs. SV shock: 15.56 ± 7.16 ml, *P* < 0.001; CI baseline: 4.24 ± 1.02 L/min vs. CI shock: 1.91 ± 0.51 L/min/m^2^, *P* < 0.001) in the hemorrhage phase. Thirty minutes after MAP recovery, SV and CI returned to baseline levels (SV baseline: 36.00 ± 6.51 ml vs. SV recovery30min: 34.22 ± 11.33 ml, *P* = 0.735; CI baseline: 4.24 ± 1.02 L/min vs. CI recovery 30 min: 3.78 ± 1.02 L/min/m^2^, *P* = 0.355).

### Correlation between CDU parameters and PiCCO parameters

**Fig. 5:**
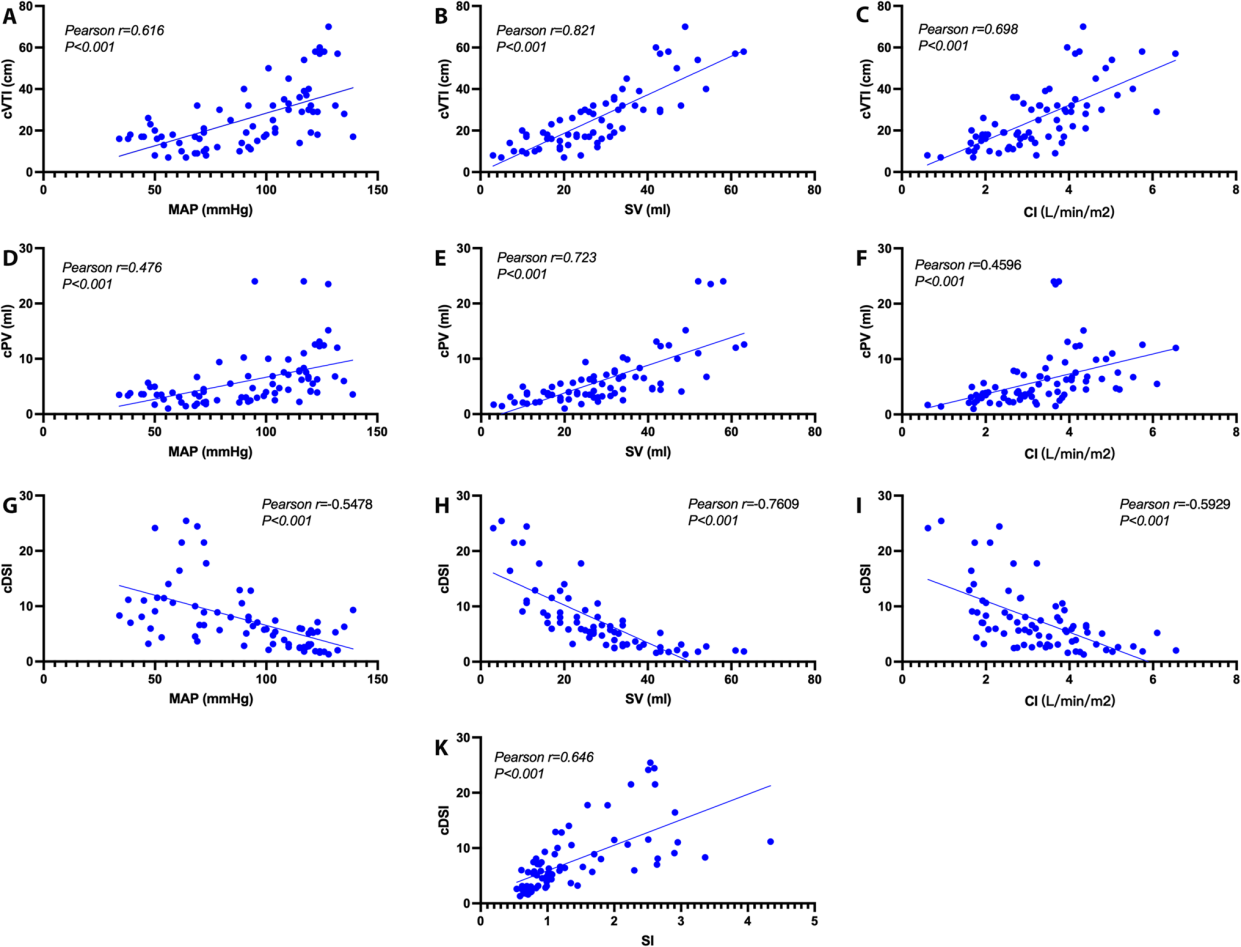
Relationship between CDU parameters and PiCCO parameters. **A** The correlation between carotid velocity time integral (cVTI) and mean arterial pressure (MAP). **B** The correlation between cVTI and stroke volume (SV). **C** The correlation between cVTI and cardiac index (CI). **D** The correlation between carotid pulse volume (cPV) and MAP. **E** The correlation between cPV and SV. **F** The correlation between cPV and CI. **G** The correlation between carotid Doppler shock index (cDSI) and MAP. **H** The correlation between cDSI and SV. **I** The correlation between cDSI and CI. **K** The correlation between cDSI and shock index (SI)

As shown in Fig. 5A–K, from the hemorrhage to fluid resuscitation stage, cVTI exhibited correlations with MAP (*r* = 0.616, *P* < 0.001) and CI (*r* = 0.698, *P* < 0.001) and a strong positive correlation with SV (*r* = 0.821, *P* < 0.001). cPV was only correlated with SV (*r* = 0.723, *P* < 0.001). cDSI was negatively correlated with MAP (*r* =  − 0.593, *P* < 0.001), SV (*r* =  − 0.761, *P* < 0.001), and CI (*r* =  − 0.548, *P* < 0.001), but positively correlated with SI (*r* = 0.647, *P* < 0.001).

## Discussion

The results of our present study provide compelling evidence that CDU parameters can offer real-time feedback on changes in MAP and exhibit correlations with invasive PiCCO monitoring parameters, suggesting that CDU monitoring could potentially replace classical invasive methods and offers the advantages of being non-invasive, feasible, real-time, and suitable for dynamic monitoring.

One of the key parameters assessed was cVTI, which reflects the blood flow through the ultrasonic section in a unit of time [11], which can be determined by tracing the pulsed-wave Doppler signal from a single heartbeat. According to the calculation formula, blood flow = cVTI × carotid area × HR, cVTI is directly related to cerebral blood flow. Our results clearly demonstrated that when MAP dropped to 60% of the baseline value, cVTI significantly decreased. This synchronous change was confirmed by the significant correlation between cVTI and MAP. Importantly, beyond numerical values, changes in the cVTI waveform pattern under different volume status conditions during the process from hemorrhage to fluid resuscitation could be objectively distinguished. In the baseline state, the cVTI waveform exhibited a wide and smooth pattern, but when there was a sharp decrease in volume, the cVTI waveform became more slender and pointed. These changes in the waveform were restored with volume compensation after fluid resuscitation. This feature facilitated rapid and non-quantitative assessment of hemorrhage in resource-limited medical conditions.

Furthermore, we found that cVTI had varying correlations with SV and CI, which are PiCCO parameters. Acute and transient changes in SV have been shown to induce proportional changes in the common carotid artery Doppler signal [12, 13]. However, it should be borne in mind that during the fluid resuscitation phase, when cVTI returned to baseline values, SV and CI remained lower than baseline, attributed to the fact that carotid blood flow depends not only on global blood flow but also on the distribution of global flow through various regional circulations. Consequently, carotid blood flow cannot be used to estimate SV or cardiac output under steady-state conditions [14, 15]. Additionally, from a device limitations perspective, cVTI exhibited high accuracy only for SV_∆_ greater than 10% [16], suggesting that while cVTI can reliably provide feedback on sudden volume reduction to monitor hemorrhagic shock, CDU needs further improvement in sensitivity during the fluid resuscitation phase.

cPV, derived from the product of cVTI and the cross-sectional area of the carotid artery, provides corresponding feedback on changes in MAP but does not exhibit a correlation between the two. This discrepancy may be due to the fact that during massive blood loss, the circulatory system relies on increasing HR to maintain the cerebral blood supply. cPV is similar to SV in that it can only reflect the volume of each stroke. Under dynamic changes in HR, it becomes challenging to establish an association between cPV and MAP. Additionally, the acquisition of cPV relies on measuring the cross-sectional area of carotid artery blood vessels using ultrasound probes, and continuous changes in blood vessel diameter may interfere with measurement accuracy.

To more accurately reflect the overall state of the circulatory system, CDU not only collects carotid blood flow data but also calculates multiple hemodynamic parameters by incorporating heart rate. One such parameter is cDSI, which is calculated from cVTI and HR collected by CDU in real time. Our research results indicate that cDSI is negatively correlated with MAP and CI, and there is a significant correlation with the traditional indicator SI (SI = HR/SBP). From this, it is highly conceivable that cDSI may become an effective warning tool for predicting shock under conditions where systolic blood pressure cannot be accurately measured.

FTc, which represents the systolic flow time corrected for pulse rate [17], has been commonly used in previous studies to reflect fluid responsiveness [18]. However, our results indicate that FTc failed to accurately provide feedback on changes in MAP at most time points, which could be attributed to the fact that various algorithmic formulas for FTc are only applicable to relatively stable heart rate, afterload, and myocardial contractility [19]. In our study, the SV of animals generally decreased by over 70% due to hemorrhage, and the rapid and massive volume loss rendered the FTc algorithm less applicable, suggesting that FTc is not a reliable volume observation indicator for severe hemorrhagic shock.

In the past decade, multiple studies have attempted to use CDU monitoring for healthy volunteers, dialysis, coronary heart disease, and cardiac surgery patients [19–22]. Under relatively stable hemodynamic conditions, CDU monitoring can reliably measure patient CO and predict the changes in intravascular volume and fluid responsiveness. In this study, when reaching the target MAP, the animal’s blood loss volume is 22.8 to 58.3% of the whole blood volume (approximately 8% of weight). This broad range of blood loss indicated the self-compensation of circulatory system for acute blood loss is a complex and dynamic process, according to our results, the effectiveness and sensitivity of some CDU parameters in intense volume changes still need further discussion.

To our knowledge, this is the first instance in which CDU has been used to monitor an HS model. Unlike common bleeding scenarios [7], abrupt volume changes in acute HS often lead to adaptations in the entire circulatory system, resulting in characteristic changes in hemodynamic parameters. Our results clearly demonstrated that PiCCO can provide more precise information regarding volume status as the gold standard, whether in hemorrhage or fluid resuscitation. As a non-invasive monitoring method, the accuracy of CDU may not be optimal, but some parameters still contribute to the diagnosis of shock and can provide non-quantitative feedback. Thus, combined with its unique easy operability, CDU may become a feasible method for monitoring and guiding fluid resuscitation in acute hemorrhagic shock under resource-limited medical conditions.

Our study has several limitations that should be addressed in future investigations. Firstly, the small sample size could have resulted in type 1 error. To mitigate this limitation, efforts were undertaken to use PiCCO systems as hemodynamic monitors to ensure experiment quality. Secondly, this study included only female animals to minimize heterogeneity. Future studies should include both male and female animals to explore the role of sex in susceptibility to shock. Doppler velocity measurements are also subject to human error. Factors such as an incorrect Doppler angle, sample volume, or Doppler gain settings may lead to measurement inaccuracies.

## Conclusion

In conclusion, compared with PiCCO, CDU monitoring can reliably reflect the volume status of HS and fluid resuscitation. It possesses the characteristics of being non-invasive, real-time, and simple to operate, which aids in the assessment and treatment of hemorrhagic shock under resource-limited conditions.

## Data Availability

The authors confirm that the data supporting the findings of this study are available within the article.

## References

[1] Lozano R, Naghavi M, Foreman K, et al. Global and regional mortality from 235 causes of death for 20 age groups in 1990 and 2010: a systematic analysis for the Global Burden of Disease Study 2010. Lancet. 2012;380:2095–128. 10.1016/S0140-6736(12)61728-0. 23245604 10.1016/S0140-6736(12)61728-0 PMC10790329

[2] Halmin M, Chiesa F, Vasan SK, et al. Epidemiology of massive transfusion: a bi-national study from Sweden and Denmark. Crit Care Med. 2016;44:468–77. 10.1097/CCM.0000000000001410. 26901542 10.1097/CCM.0000000000001410

[3] Mitra B, Gabbe BJ, Kaukonen K-M, Olaussen A, et al. Longterm outcomes of patients receiving a massive transfusion after trauma. Shock. 2014;42:307–12. 10.1097/SHK.0000000000000219. 24978897 10.1097/SHK.0000000000000219

[4] Litton E, Morgan M. The PiCCO monitor: a review. Anaesth Intensive Care. 2012;40(3):393–409. 10.1177/0310057X1204000304. 22577904 10.1177/0310057X1204000304

[5] Faldaas BO, Nielsen EW, Storm BS, et al. Hands-free continuous carotid Doppler ultrasound for detection of the pulse during cardiac arrest in a porcine model. Resusc Plus. 2023;20(15):100412. 10.1016/j.resplu.2023.100412. 10.1016/j.resplu.2023.100412 PMC10336194 37448689

[6] Hossein-Nejad H, Mohammadinejad P, Lessan-Pezeshki M, et al. Carotid artery corrected flow time measurement via bedside ultrasonography in monitoring volume status. J Crit Care. 2015;30(6):1199–203. 10.1016/j.jcrc.2015.08.014. 26410681 10.1016/j.jcrc.2015.08.014

[7] Karadadaş S, Çorbacıoğlu ŞK, Çevik Y, et al. Assessment of the carotid artery Doppler flow time in patients with acute upper gastrointestinal bleeding. Turk J Emerg Med. 2020;20(1):35–41. 10.4103/2452-2473.276387. 32355900 10.4103/2452-2473.276387 PMC7189818

[8] Barjaktarevic I, Toppen WE, Scott H, et al. Ultrasound assessment of the change in carotid corrected flow time in fluid responsiveness in undifferentiated shock. Crit Care Med. 2018;46(11):1040–6. 10.1097/CCM.0000000000003356. 10.1097/CCM.0000000000003356 PMC6774608 30134304

[9] World Medical Association Declaration of Helsinki. ethical principles for medical research involving human subjects. JAMA. 2013;310(20):2191–4. 10.1001/jama.2013.281053. 24141714 10.1001/jama.2013.281053

[10] Alexander Ziebart, Jens Kamuf, Robert Ruemmler, et al.Standardized hemorrhagic shock induction guided by cerebral oximetry and extended hemodynamic monitoring in pigs. J Vis Exp. 2019;(147). 10.3791/59332. 10.3791/59332 31180364

[11] Suriani I, van Houte J, de Boer EC, et al. Carotid Doppler ultrasound for non-invasive haemodynamic monitoring: a narrative review. Physiol Meas. 2023;43(10):1001. 10.1088/1361-6579/ac96cb. 10.1088/1361-6579/ac96cb 36179705

[12] Sidor M, Premachandra L, Hanna B, et al. Carotid flow as a surrogate for cardiac output measurement in hemodynamically stable participants. J Intensive Care Med. 2018;1:885066618775694. 10.1177/0885066618775694. 10.1177/0885066618775694 29742951

[13] Jalil B, Thompson P, Cavallazzi R, et al. Comparing changes in carotid flow time and stroke volume induced by passive leg raising. Am J Med Sci. 2018;355:168–73. 10.1016/j.amjms.2017.09.006. 29406045 10.1016/j.amjms.2017.09.006

[14] Weber U, Glassford NJ, Eastwood GM, et al. A pilot assessment of carotid and brachial artery blood flow estimation using ultrasound Doppler in cardiac surgery patients. J Cardiothorac Vasc Anesth. 2016;30:141–8. 10.1053/j.jvca.2015.06.025. 26411812 10.1053/j.jvca.2015.06.025

[15] Weber U, Glassford NJ, Eastwood GM, et al. A pilot study of the relationship between Doppler-estimated carotid and brachial artery flow and cardiac index. Anaesthesia. 2015;70:1140–7. 10.1111/anae.13069. 26010229 10.1111/anae.13069

[16] Kenny JS, Barjaktarevic I, Mackenzie DC, et al. Carotid artery velocity time integral and corrected flow time measured by a wearable Doppler ultrasound detect stroke volume rise from simulated hemorrhage to transfusion. BMC Res Notes. 2022;15(1):7. 10.1186/s13104-021-05896-y. 35012624 10.1186/s13104-021-05896-y PMC8750810

[17] Mackenzie DC, Khan NA, Blehar D, et al. Carotid flow time changes with volume status in acute blood loss. Ann Emerg Med. 2015;66:277–82. 10.1016/j.annemergmed.2015.04.014. 26003002 10.1016/j.annemergmed.2015.04.014

[18] Beier L, Davis J, Esener D, et al. Carotid ultrasound to predict fluid responsiveness: a systematic review. J Ultrasound Med. 2020;10:1965–76. 10.1002/jum.15301. 10.1002/jum.15301 32314817

[19] Kenny JS, Barjaktarevic I, Mackenzie DC, et al. Diagnostic characteristics of 11 formulae for calculating corrected flow time as measured by a wearable Doppler patch. Intensive Care Med Exp. 2020;8(1):54. 10.1186/s40635-020-00339-7. 32940808 10.1186/s40635-020-00339-7 PMC7498524

[20] Peng QY, Zhang LN, Ai ML, et al. Common carotid artery sonography versus transthoracic echocardiography for cardiac output measurements in intensive care unit patients. J Ultrasound Med. 2017;9:1793–9. 10.1002/jum.14214. 10.1002/jum.14214 28429475

[21] Hossein-Nejad H, Mohammadinejad P, Lessan-Pezeshki M, et al. Carotid artery corrected flow time measurement via bedside ultrasonography in monitoring volume status. J Crit Care. 2015;30(6):1199–203. 10.1016/j.jcrc.2015.08.014. 26410681 10.1016/j.jcrc.2015.08.014

[22] van Houte J, Raaijmaakers AE, Mooi FJ, et al. Evaluating corrected carotid flow time as a non-invasive parameter for trending cardiac output and stroke volume in cardiac surgery patients. J Ultrasound. 2023;1:89–97. 10.1007/s40477-022-00678-z. 10.1007/s40477-022-00678-z PMC10063698 35397758

